# SILC for SILC: Single Institution Learning Curve for Single-Incision Laparoscopic Cholecystectomy

**DOI:** 10.1155/2013/381628

**Published:** 2013-05-09

**Authors:** Chee Wei Tay, Liang Shen, Mikael Hartman, Shridhar Ganpathi Iyer, Krishnakumar Madhavan, Stephen Kin Yong Chang

**Affiliations:** ^1^Department of Surgery, Division of Hepatobiliary and Pancreatic Surgery, National University Health System, Singapore 119228; ^2^Division of Biostatistics, Yong Loo Lin School of Medicine, National University of Singapore, Singapore 119228; ^3^Department of Medical Epidemiology and Biostatistics, Karolinska Institute, 17177 Stockholm, Sweden; ^4^Saw Swee Hock School of Public Health, National University of Singapore, Singapore 117597

## Abstract

*Objectives*. We report the single-incision laparoscopic cholecystectomy (SILC) learning experience of 2 hepatobiliary surgeons and the factors that could influence the learning curve of SILC. *Methods*. Patients who underwent SILC by Surgeons A and B were studied retrospectively. Operating time, conversion rate, reason for conversion, identity of first assistants, and their experience with previous laparoscopic cholecystectomy (LC) were analysed. CUSUM analysis is used to identify learning curve. *Results*. Hundred and nineteen SILC cases were performed by Surgeons A and B, respectively. Eight cases required additional port. In CUSUM analysis, most conversion occurred during the first 19 cases. Operating time was significantly lower (62.5 versus 90.6 min, *P* = 0.04) after the learning curve has been overcome. Operating time decreases as the experience increases, especially Surgeon B. Most conversions are due to adhesion at Calot's triangle. Acute cholecystitis, patients' BMI, and previous surgery do not seem to influence conversion rate. Mean operating times of cases assisted by first assistant with and without LC experience were 48 and 74 minutes, respectively (*P* = 0.004). *Conclusion*. Nineteen cases are needed to overcome the learning curve of SILC. Team work, assistant with CLC experience, and appropriate equipment and technique are the important factors in performing SILC.

## 1. Introduction

Single-incision laparoscopic cholecystectomy (SILC) has been increasingly performed for benign gallbladder disease over the last few years with comparable operative results with conventional 4-port laparoscopic cholecystectomy (CLC). With results from randomized controlled trials (RCTs) [1–5] and series of publications [6–9] showing that SILC is equally safe, with no obvious additional scar and potentially have less postoperative pain and earlier return to daily activity [5], more surgeons are embarking on learning the technique.

As SILC is a new approach to gallbladder disease, many aspects of this new technique have not been studied in detail. Most surgeons embarking on this technique are concerned with its learning curve, conversions, and potential longer operating time. To date, very limited work has been done to look into this important issue and few publications have looked into learning curve of SILC from conversion point of view. 

To perform SILC safely and successfully, there may be changes in surgical technique, need of new equipment, and modifications in the role of assistant. 

In this study, we report an SILC learning experience of a tertiary university hospital with advanced laparoscopic facility. Operating time, potential problems, and ways to overcome them as well as surgical technique were included in this report. Our paper aims at facilitating and smoothening the learning curve of surgeons especially those who are starting to perform SILC or those facing difficulty in performing SILC.

## 2. Methods

All patients who underwent SILC from April 2009 to August 2011 (28 months) by two HPB attending surgeons (Surgeons A and B) who both have been attending grade for more than 7 years and routinely performed laparoscopic cholecystectomy for all benign gallbladder disease in a tertiary university hospital were studied retrospectively. The unit performs about 400 laparoscopic cholecystectomies per year.

Operating time, conversion rate, and reason for conversion of individual surgeons were recorded. Conversion is defined as adding additional port(s) at other parts of the abdomen or minilaparotomy. Identity of first assistants was collected and analysed. Risk factors of conversion such as patient's BMI, presence of acute cholecystitis, and previous abdominal surgery were recorded and compared.

Cumulative summative (CUSUM) analysis is used to identify learning curve of SILC of Surgeon A, and standard conversion rate is defined as 5%. *t*-test is used to compare continuous variable, and *P* < 0.05 is defined as statistical significance. SPSS Statistics version 17.0 is used to analyse the data.

Operating time of all CLC done by Surgeon A at the same period of time was collected to establish the baseline operating time for comparison with SILC operating time of Surgeons A and B.

### 2.1. SILC Surgical Methods

**Figure 1:**
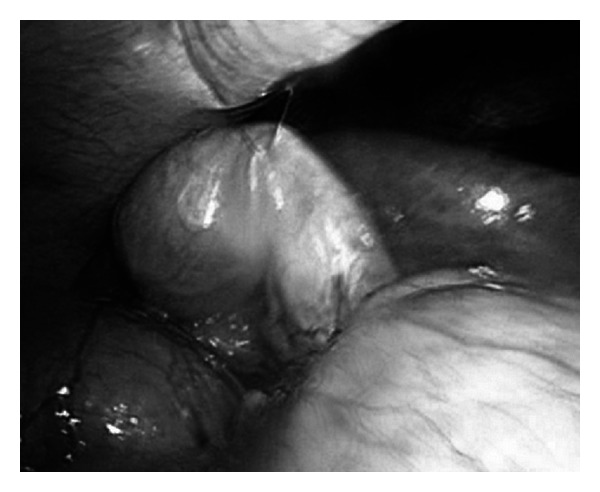
Hanging suture place at gallbladder fundus.

All procedures were performed under general anaesthesia. The patients were placed at supine or split-leg (French) position depends on availability of different operating tables. Marcaine 0.25% is infiltrated around the umbilicus then a 1.5 cm vertical incision is made in the umbilicus, and SILS port (Covidien, Dublin, Ireland) is then inserted. A 5 mm 30° Endo-EYE surgical videoscope (Olympus, Tokyo, Japan) is used for visualization of the entire operation. Prolene suture with straight needle is introduced percutaneously at the right hypochondrium and is made to pierce the gallbladder at the seromuscular plane before exiting the peritoneal cavity at the right hypochondrium (Figure 1); care is taken not to pierce through the mucosa to prevent bile spillage. This serves as a retraction suture to facilitate the exposure of the Calot's triangle and subsequent dissection.

**Figure 2:**
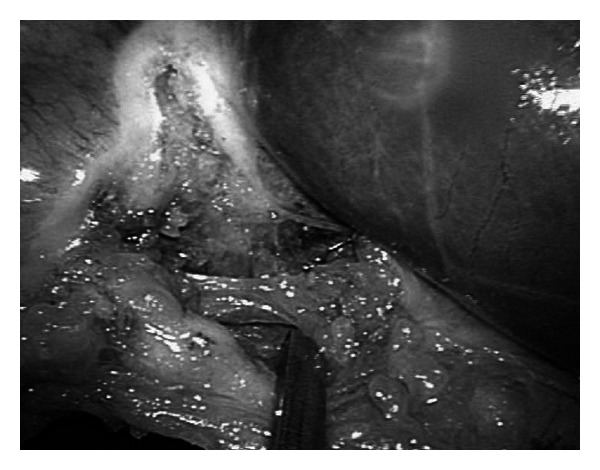
Articulating forcep used to retract Hartmann's pouch to expose Calot's triangle and critical view of safety is visualized.

An articulating endoforcep, Roticulator (Covidien, Dublin, Ireland), is introduced to provide lateral retraction of the gallbladder, and careful dissection to achieve critical view of safety is then completed (Figure 2). 

**Figure 3:**
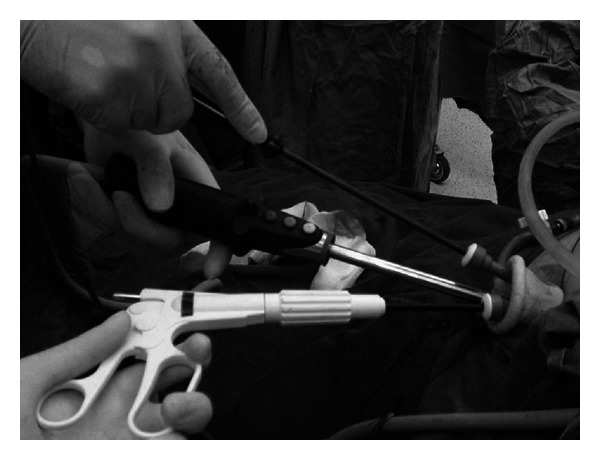
“Snooker cue guide” position.

Both the surgeon and the assistant will be on the patient's left if the patient is on supine position, whereas the operating surgeon will be standing between patient's legs and the assistant will be on the patient's left side if the patient is on split-leg position. The assistant would sit in front of the surgeon. In most parts of the surgery, he will be providing gentle lateral traction of the gallbladder by manipulating the Roticulator while the primary surgeon holds the EndoEYE and the dissecting instruments in the “snooker cue guide” position (Figure 3). This position allows the camera and the dissecting instrument to move in a coordinated fashion to ensure optimal visualization of the dissecting process which is critical in safely exposing the Calot's triangle to identify the cystic artery and duct. Five mm Hem-o-lock (Teleflex Medical, USA) clips are used to ligate both cystic artery and duct before they are divided between clips. Gallbladder is then placed into a self-constructed bag intracorporeally and removed from the abdominal cavity; fascia is closed with nonabsorbable suture in figure-of-eight fashion, and skin is closed subcuticularly.

## 3. Results

One hundred and nineteen patients who underwent SILC for their gallbladder diseases between April 2009 and August 2011 by 2 HPB consultants (Surgeons A and B) were retrospectively studied. One hundred and nineteen cases were performed by Surgeons A and B, respectively. 7 (5.8%) cases were acute cholecystitis and 75 cases (94.1%) were chronic cholecystitis. Diagnosis of gallbladder disease was achieved by clinical information and pre-op radiological investigations (ultrasound scan or CT scan). There were 8 cases (6.7%) that needed extra working port(s) to complete the procedure; no open conversion was needed in our experience.

### 3.1. Learning Curve of SILC

**Figure 4:**
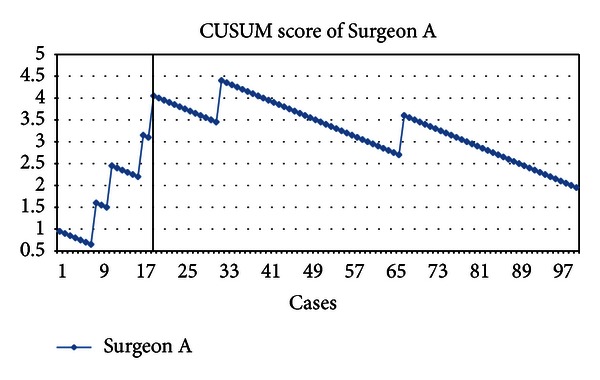
CUSUM analysis of learning curve of Surgeon A.

We defined acceptable conversion rate of SILC as 5% after learning curve is overcome as this is considered traditionally an acceptable conversion rate in CLC. Surgeons A and B had 6 (6%) and 2 (10.9%) conversions respectively.  Figure 4 shows the CUSUM analysis of learning curve of Surgeon A; vertical line at the 19th case indicates the predicted minimal number of cases required to overcome the SILC learning curve. Surgeon B is excluded from CUSUM analysis in this study due to limited number of cases performed.

Most conversions of Surgeon A happened before the first 19 cases, and subsequently his learning curve reached a plateau except two conversions in the 32nd and 67th case. Surgeon B had two conversions in his 1st and 4th case. Most conversions were due to dense adhesion at the Calot's triangle and vital anatomical structures cannot be visualized clearly. One (5%) patient with previous abdominal surgery required conversion and one (5%) patient with active acute cholecystitis required conversion.  Table 1 shows the operative and patient profile of the first 19 cases of Surgeons A and B.  Table 2 shows the profile of cases that required conversion in the first 19 cases. When comparing cases which required conversion and cases which did not require conversion, there is no significant difference between patients (1) with previous, without previous, or on-going acute cholecystitis, (2) previous abdominal surgery, and (3) mean BMI.  Table 3 demonstrates the comparison of potential risk factors between cases with and without conversion.

### 3.2. Operating Time

Surgeon A's mean operating time is significantly lower (62.5 minutes versus 90.6 minutes, *P* = 0.04) after he has overcome the learning curve. Conversion rates were lower as well (2.5% versus 21%, *P* = 0.36). Mean operating times, conversion rate, and patients' profile of Surgeons A before and after the first 19 cases is shown in Table 4. 

**Figure 5:**
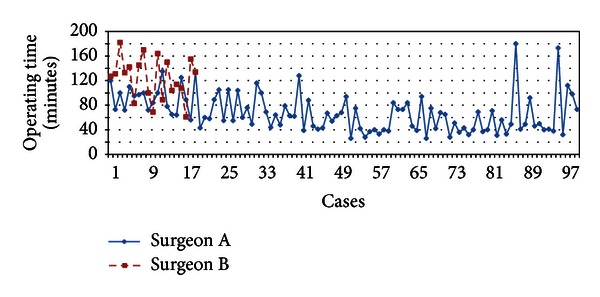
Operating times of Surgeons A and B.

**Figure 6:**
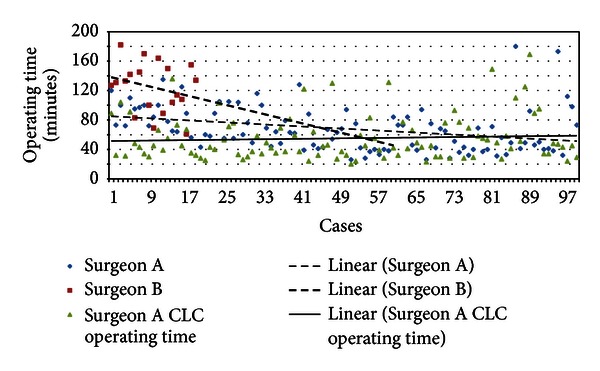
Trend lines of operating time of Surgeons A and B. Trend line of Surgeon B showed faster improvement in operating time with mentoring from Surgeon A.

Figure 5 demonstrates the operating times of Surgeons A and B as their experience increased.  Figure 6 demonstrates the trend line of operating time of Surgeon A (dashed line) and B (dotted line). We found that the trend line of operating time of Surgeon B is steeper than Surgeon A, hence suggests that guidance from another surgeon who is experienced in SILC can facilitate the learning curve rapidly. Surgeon A SILC operating time trend line crosses his CLC operating time trend line (straight line) at the 82th case, which is suggestive of that SILC operating time may be faster than CLC eventually as the experience increases further.

**Figure 7:**
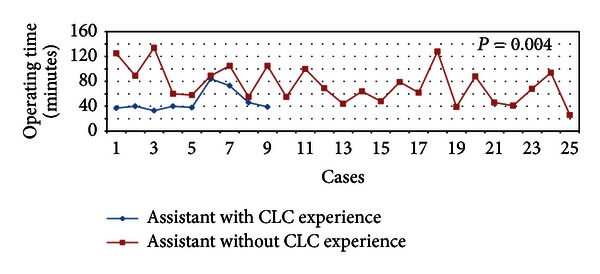
Operating time of cases assisted by assistants with and without CLC experience.

We compared the 2 HPB fellows who have assisted in most of the SILC cases of Surgeon A in our institution, one who had previous CLC experience and the other without. We found that the mean operating time of cases assisted by the assistant with CLC experience is significantly shorter in comparison with cases assisted by the assistant without previous CLC experience (48 versus 74 minutes, *P* = 0.004). Mean operating time of cases assisted by the 2 assistants and the trend are demonstrated in Table 5 and Figure 7 respectively.

## 4. Discussion

### 4.1. Operating Time and Conversion

Our studies demonstrated that the operating time of SILC was more than 90 minutes at the beginning of both surgeons. Surgeon A was able to achieve mean operating time of below 60 minute after about 50 cases of SILC and his mean operating time continues to decrease to 37 minutes after 60 cases. Antoniou et al. [10] reported that the mean operative time was 70.2 minutes in a systemic review which involved 29 studies with 1166 patients. However, most of the studies included were the early experiences of surgeon performing SILC in their individual centres. In comparison, our studies showed that mean operative time continues to decrease as experiences increase after the learning curve is overcome. Other publications [11–13] that looked into SILC operative time and learning curve reported a mean operative time between 46.9 minutes and 80 minutes. Hernandez et al. [11] found that mean operative time was reduced significantly after 75 cases of SILC and was not significantly longer than mean operative time of CLC. Our institution showed similar studies data. Qiu et al. [12] reported a much shorter mean operative time of 46.9 minutes with no conversion in their highly selected 80 patients, all of whom have minimal sign of gallbladder inflammation and no surgical history of the right upper quadrant of abdomen. They were able to perform SILC with mean operative time of below 40 minutes after 40 cases. Joseph et al. [14] concluded that surgical trainees who were proficient in CLC had significant reduction in operative time along their learning curve. Recently published RCTs [1–5] reported mean operative time between 46 minutes and 88 minutes with 3 studies [1, 2, 4] which showed significant longer operative time of SILC; however, these RCTs did not specify the surgeons' previous CLC and SILC experience and all of them did not include patients with acute cholecystitis. 

There were 8 (6.7%) cases in our studies which required additional port(s) to aid dissection of the Calot's triangle due to dense adhesion at the area; no open conversion or laparotomy was needed in our studies. Four (80%) out of the 5 conversions of Surgeon A happened before his first 20 cases. Surgeon B had two conversions at his 1st and 7th case. The systemic review published by Antoniou et al. [10] reported a conversion (additional ports required) rate of 9.3% and an open conversion rate of 0.4%. Most common conversion reason that was reported was an obscured anatomy of the Calot's triangle due to adhesions, acute or chronic inflammation (71.1%). Seven out of 8 (87%) of our conversions were due to severe adhesions at the Calot's triangle as well. In conclusion, our study was found to have very similar rate and reason of conversion with Antoniou's study [10].

One of our conversions was associated with previous abdominal surgery. However, the reason for inserting an additional port was to place a clip at a leaking cystic duct. Hence, we do not think that the previous abdominal surgery has any significance on this conversion. In another conversion which was associated with an on-going acute cholecystitis, two additional ports were added to provide retraction for adequate visualization as well as to secure haemostasis from the liver bed. We performed SILC on 4 other cases of acute cholecystitis with no significant issues.

In our center, Surgeon A was the first HPB surgeon who adopted SILC into his routine treatment option for gallbladder diseases, followed by Surgeon B. From our CUSUM analysis, Surgeon B had less conversion in the early stages of his SILC learning curve in comparison to Surgeon A. Hence, we deduced that during the process of pioneering this new surgical technique in our center, Surgeon A inevitably had more conversions than other surgeons in the center before his learning curve was overcome. 

Once the expertise is shared among other surgeons, we would expect less conversion and smoother learning curve in the subsequent cases. This phenomenon was demonstrated in the steeper trend line of operating time of Surgeon B, after Surgeon A has overcome his learning curve of SILC. With less skin incisions in SILC hence less closure time, we believe the operating time could be faster than CLC eventually as the experience increases, as shown in our results.

Analyzing the CUSUM, significantly less conversion was experienced after the 19th case; we therefore conclude that surgeons who routinely perform CLC for gallbladder diseases need about 19 cases to overcome SILC learning curve.

### 4.2. Assistant Factor

In the beginning phase of adopting new surgical technique or equipment in our center, we found that there are always benefits if the same group of surgeons and nurses can provide feedbacks among themselves to hasten the learning process. 

We compared the operating times with 2 HPB fellows as assistants; one routinely performs CLC in her practice and one was new to CLC; both were new to SILC. We found that there was significant shorter mean operating time in cases that were assisted by the fellow who was familiar with CLC. SILC is a procedure that requires advanced laparoscopic skills. In addition, the surgeon and his/her assistant must be able to work closely with each other in a more limited space without colliding their instruments against each other. In addition, having CLC experience prior to assisting SILC is an invaluable advantage. Qiu et al. [12] and Solomon et al. [13] both had similar learning experience, and hence they encouraged surgeons to work with skilled assistant and obtaining preceptorship in order to overcome one's SILC learning curve.

We also encouraged other surgeons to record a video of all their SILC cases, and subsequently watch the video together with their assistant, with the aim of identifying weaknesses and mistakes and avoid them in subsequent cases. 

### 4.3. Technique and Equipment Issues

In SILC, all surgical equipment is introduced from the umbilical port site. Manipulation of the instruments intra- and extracorporeally is thus very challenging due to the limited working space and loss of the traditional laparoscopic triangulation. We started our SILC practice with SILS port as intraperitoneal access, it accommodates all working instruments, insufflation and camera port, and is inserted through a single fascial defect. This port does increase the cost of surgery, however in our experience, there is no significant surgical or technical problems caused by the port, and we continued to improve our operating time and conversion rate with the help of this port; therefore, it remains as the port of choice for intraperitoneal access. 

In order to overcome the loss of laparoscopic triangulation, we utilized the Roticulator forceps, which is held by the first assistant, who sits at the right side of the surgeon. The forceps provide lateral retraction of the gallbladder to facilitate the dissection of Calot's triangle. We realized that with SILS surgery, especially in someone who just started performing SILS surgery, loss of conventional triangulation in manipulating the instruments and loss of working space can be frustrating to the surgeons and dangerous to the patients; we recommend surgeons who are new to SILC to use articulating or prebend instruments to facilitate the surgery in the first few cases of SILC, and with the increased experience in SILC, they can make a choice to continue in using these instrument or switch to conventional laparoscopic instruments. Again, these articulating or pre-bend instruments add extra cost to the patients; however, in view of the advantages mentioned above, we believe it has an important role in SILC, especially in those surgeons who are new to SILC.

The other equipment which we found to be of value is the Olympus Endoeye, which is a very compact and highly manipulable laparoscopic camera that provides adequate visualization for the scope of SILC surgery without occupying much space.

We routinely used extracorporeal hanging suture to enhance the visualization of SILC. In this way, 2 instruments can actively be used in performing the surgery. We manipulate the straight needle laparoscopically and pierce the thickest part of the gallbladder at the seromuscular layer, to prevent bile spillage; so far, there is no issue in all the cases we performed in this series. In addition, hanging suture has been shown to reduce complication rates in comparison with instrumental anchorage [10] (3.3% versus 13.3%, *P* < 0.0001).

Port site hernia has been a concern in SILC due to the bigger umbilical fascial defect if compared to CLC, a 52-patient retrospective study [15] published a port site hernia rate of SILC of 5.8%. Multiple up-to-date meta-analysis [16–18] has not shown significant increase in port site hernia so far; the majority of the RCTs performed up-to-date utilized commercialized umbilical access port, and these studies are limited with their short follow-up period. Goel and Lomanto [19] concluded in their review that port site hernia in single-incision laparoscopic surgery can be minimized with good suture closure of the fascial defect. We close all umbilical fascial defects with 1 or 2 figure-of-eight sutures; there is no umbilical hernia detected in this series of patients during followup.

### 4.4. Patient Selection

Patients with risk factors such as previous abdominal surgery, history of acute cholecystitis or on-going cholecystitis and obese patient were thought to have higher chance of conversion in SILC [10]. However in our experience, all of our patients who needed conversion to CLC, did not evidently presented with the above risk factors. In fact, the most common reason for conversion was dense adhesions and failure to identify vital structures due to poor visualization. Patients with the above risk factors are shown to increase operative time [12], therefore we suggest selecting patients sensibly at the early stage of performing SILC. Once our learning curve has been overcome, we were able to perform SILC in majority of the gallbladder condition in the general patient population with minimal conversion rate.

## 5. Conclusion

Single-incision laparoscopic cholecystectomy is a safe and feasible procedure. Nineteen cases were needed to overcome the learning curve in our experience. Comparable conversion rate and operating time with conventional laparoscopic cholecystectomy were observed after learning curve has been overcome. Team work, careful patient selection, assistant with conventional laparoscopic cholecystectomy experiences, and appropriate equipment and technique are important factors at the beginning stage of performing SILC.

## Figures and Tables

**Table 1:** Operative and patient profile of the first 19 cases of Surgeons A and B.

	Surgeon A	Surgeon B
Cases, *n*	19	19
Mean operative time, minutes (range, SD)	90.6 (43–135, 25.8)	124.3 (61–182, 34.1)
Conversion rate, *n* (%)	4 (21%)	2 (11%)
Acute cholecystitis, *n* (%)	3 (16%)	1 (5%)
Previous abdominal surgery, *n* (%)	3 (16%)	1 (5%)
Mean BMI (range, SD)	25.4 (19.2–36.0, 4.8)	22.4 (16.0–30.5, 4.0)

**Table 2:** Profile of cases that required conversion in the first 19 cases.

Patient	Surgeon A/B	Reason	Types of conversion	Previous or on-going acute cholecystitis	Previous abdominal surgery	BMI (kg/m^2^)	Operating time (minutes)
1	A	Bile leak from cystic duct	1 × additional 5 mm port	No	Yes	22.6	100
2	A	Dense adhesion at Calot's triangle	1 × additional 5 mm port	No	No	28.3	100
3	A	Acute cholecystitis with dense adhesion at Calot's triangle and gallbladder bed bleeding	2 × additional 5 mm ports	Yes	No	27.0	89
4	A	Gallbladder densely adherent to liver	2 × additional 5 mm ports	No	No	25.0	134
5	B	Dense adhesion at Calot's triangle	3 × additional 5 mm ports	No	No	29.8	127
6	B	Dense adhesion at Calot's triangle	2 × additional 5 mm ports	No	No	21.8	145

**Table 3:** Comparison of potential risk factors in cases with and without conversion.

	Cases required conversion	Cases did not require conversion	*P*
*n*	8	111	—
On-going or previous acute cholecystitis, *n* (%)	1 (13%)	9 (8%)	0.63
Previous abdominal surgery, *n* (%)	0 (0%)	6 (5%)	0.06
Mean BMI (range, SD)	25.8 (21.8–29.8, 3.2)	24.1 (17.5–36, 4.6)	0.13

**Table 4:** Mean operating times, conversion rate, and patients' profile of Surgeons A after the first 19 cases.

	Surgeon A's subsequent 81 cases	Surgeon A's first 19 cases	*P*
Cases, *n*	81	19	
Operative time, minutes (range, SD)	62.5 (26–180, 30.2)	90.6 (43–135, 25.8)	**0.04**
Conversion rate, *n* (%)	2 (2.5%)	4 (21%)	0.36

**Table 5:** Mean operating time of cases assisted by assistants with and without CLC experience.

	Mean operating time (minute)	Max (minute)	Min (minute)	SD (±)	*P*
Assistant with CLC experience	48	84	33	18	**0.004**
Assistant without CLC experience	74	134	26	29	

## Abbreviations

SILC: Single-incision laparoscopic cholecystectomy
CLC: Conventional laparoscopic cholecystectomy
RCT: Randomized controlled trial
HPB: Hepatopancreatobiliary
CT: Computer tomography
CUSUM: Cumulative summative
SD: Standard deviation
BMI: Body mass index.
